# The Libyan civil conflict: selected case series of orthopaedic trauma managed in Malta in 2014

**DOI:** 10.1186/s13049-015-0183-2

**Published:** 2015-11-20

**Authors:** Colin Ng, Max Mifsud, Joseph N. Borg, Colin Mizzi

**Affiliations:** Trauma and Orthopaedics Departmental Secretary, Department of Trauma and Orthopaedics, Mater Dei Hospital, Triq Dun Karm, MSD 2090 Msida, Malta; Department of Surgery, Mater Dei Hospital, Msida, Malta

**Keywords:** Orthopaedics, Damage control surgery, Damage control orthopaedics (DCO), Improvised explosive device (IED), Blast injury, War trauma, Gustilo-Anderson, Indice de gravité simplifié 2 (IGS2)

## Abstract

**Aim:**

The purpose of this series of cases was to analyse our management of orthopaedic trauma casualties in the Libyan civil war crisis in the European summer of 2014. We looked at both damage control orthopaedics and for case variety of war trauma at a civilian hospital. Due to our geographical proximity to Libya, Malta was the closest European tertiary referral centre. Having only one Level 1 trauma care hospital in our country, our Trauma and Orthopaedics department played a pivotal role in the management of Libyan battlefield injuries. Our aims were to assess acute outcomes and short term mortality of surgery within the perspective of a damage control orthopaedic strategy whereby aggressive wound management, early fixation using relative stability principles, antibiotic cover with adequate soft tissue cover are paramount. We also aim to describe the variety of war injuries we came across, with a goal for future improvement in regards to service providing.

**Methods:**

Prospective collection of six interesting cases with severe limb and spinal injuries sustained in Libya during the Libyan civil war between June and November 2014.

**Conclusions:**

We applied current trends in the treatment of war injuries, specifically in damage control orthopaedic strategy and converting to definitive treatment where permissible. The majority of our cases were classified as most severe (Type IIIB/C) according to the Gustilo-Anderson classification of open fractures. The injuries treated reflected the type of standard and improved weaponry available in modern warfare affecting both militants and civilians alike with increasing severity and extent of damage. Due to this fact, multidisciplinary team approach to patient centred care was utilised with an ultimate aim of swift recovery and early mobilisation. It also highlighted the difficulties and complex issues required on a hospital management level as a neighbouring country to war zone countries in transforming care of civil trauma to military trauma.

## Introduction

Malta has been closely involved in the Libyan civil conflict on a geographical, political and humanitarian level since its inception around the year 2011, also commonly referred to as the ‘Arab Spring’. Due to our geographical proximity, as one of Libya’s closest European neighbouring countries, Malta received both civilian refugees and military casualties of war. As of the European summer of 2014, Malta has been receiving polytrauma war casualties evacuated by air and sea after initial damage control surgery and medical stabilisation of battlefield injuries in Libya. These casualties were a mixture of insurgency fighters and civilians transferred directly to our primary hospital, Mater Dei Hospital, in varying states of injury and morbidity. After being stabilised primarily by an emergency trauma surgical trauma team on the ground in Libya, they were transferred to Malta for further treatment. This involved further medical stabilisation, damage control surgery (DCS), damage control orthopaedics (DCO) and/or definite orthopaedic surgery. Our experience of being a neighbouring country to a country in civil war is paralleled to the Akkucuk et al. (2015) [1], in their paper reporting their experience from Turkey bordering the civil war stricken Syria in which a Level 1 civilian trauma centre became a military trauma centre.

The consensus through current war trauma literature is that between 65-70 % of war wounds involve the musculoskeletal system [2, 3]. The nature and thus prognosis of warfare injuries differ from general civilian orthopaedic practice [1, 4, 5] due to the dangerous environment in which the injuries are sustained, the increased severity of the injuries, the increased number of body regions involved and the staged resuscitation. The current basic war surgery principles advocated worldwide, consist of aggressive resuscitation, early and thorough debridement of the wounds, short term bridging procedures to achieve stability, then rapid evacuation to centre for definitive treatment [1, 6].

## Methods

In this series, we present six cases from a total of over one hundred and fifty trauma cases with varied musculoskeletal peripheral and spinal injuries that were treated at our Trauma and Orthopaedics department at Mater Dei Hospital in Malta. The patients in this series were collected prospectively between June 2014 and January 2015. They were brought to Malta via air ambulance and transferred directly to our general hospital during the still on-going Libyan civil war. All the patients presented here were Libyan male nationals aged from 22 to 50 years.

The time of presentation of ranged from acutely (within 3 days post injury) up to a maximum of three weeks post trauma. The patients had limb and/or spinal trauma that required damage control procedures, often with external fixation in the initial phase on the Libyan battlefield itself or district hospitals performed by Libyan surgeons.

We rarely received any accompanying documentation of the surgical procedures performed. All patients arrived at Mater Dei Hospital in varying states of haemodynamic stability. The cause of their injuries were either due to improvised explosive devices (IED), gunshot wounds (GSW), rocket propelled grenades (RPG) and/or explosive blasts with shrapnel injuries. The lower limbs were involved in four of the cases, the upper limbs in one case, and the spine in two cases. Most cases presented with comminuted open fractures; four cases presented with polytrauma needing intervention by other surgical specialities concurrently (see Table 1). Table 2 shows progression from initial fixation through to final outcome.

**Table 1:** Table showing the six patients presented in this series. All patients were male

Case	Age	Aetiology	Bone injuries	Associated injuries
1	40	IED, blast injury	Left subtrochanteric femoral fracture, right open ankle fracture, missing calcaneum (Gustilo-Anderson type IIIC)	Posterior parietal soft tissue contusion, right lower limb traumatic vascular dysfunction, sepsis
2	50	IED and GSW	Right open elbow fracture (Gustilo-Anderson type IIIB), left open comminuted mid-shaft humeral fracture (Gustilo-Anderson type IIIC)	Brachial and ulnar artery erosion, multiple metallic foreign bodies
3	32	GSW/RPG	Left comminuted proximal femur fracture (Gustilo-Anderson type IIIA), right open tibia/fibular fracture (Gustilo-Anderson type IIIB), right superior and inferior pubic rami fracture, T12 vertebral body fracture	Neurological compromise left leg with sciatic nerve palsy, bilateral lung contusion
4	22	Direct GSW	Right comminuted open knee complex fracture (Gustilo-Anderson type IIIB)	Neurological status right leg impaired, but vascular status leg intact
5	26	IED/Car bomb	T5 metal foreign body, right scapular and rib fracture	Paraplegia, left pneumothorax, multiple metallic foreign bodies, Deep venous thrombosis left leg
6	27	Above ground explosive blast	Right lateral four ray traumatic amputation, extensive soft tissue loss lateral aspect of leg	Loss of sensation along superficial peroneal nerve distribution. No other significant injuries

Age, primary injuries and associated injuries are tabulated here

**Table 2:** Summary of the interventions performed and outcomes achieved

Case	Bone injuries	Primary fixation	Secondary fixation	Outcome
1	Left subtrochanteric femoral fracture, Right open ankle fracture, missing calcaneum	Left femoral external fixator, right tibio-metatarsal external fixator	Left Intramedullary femoral nail, right below knee amputation	Transferred to rehabilitation hospital
2	Right open elbow fracture, left comminuted mid-shaft humeral fracture	Right humero-ulnar external fixator and multiple Kirsches wire fixation, left non-spanning humeral external fixator	Repeated soft tissue debridements and necrectomies, removal of infected metalwork	Inpatient mortality due to sepsis
3	Left comminuted proximal femur fracture, right open tibia/fibular fracture, right superior and inferior pubic rami fracture, T12 vertebral body fracture	Left femoral external fixator, right tibio-calcaneal external fixator	Left Intramedullary femoral nail, right conversion to ring external fixator	Transferred to rehabilitation hospital
4	Right comminuted open distal femur and tibial fracture	Right femoro-tibial external fixator	Repeated soft tissue debridements and necrectomies, Planned knee fusion	Discharge against medical advise
5	T5 metal foreign body, Right scapular and rib fracture	Nil	Observations, Acute rehabilitation	Transferred to rehabilitation hospital
6	Traumatic amputation of lateral four metatarsals of right foot	Right tibio-metatarsal external fixator	Repeated soft tissue debridements then eventual right below knee amputation as foot deemed unsalvageable	Transferred to rehabilitation hospital

The primary fixation was performed in Libya, and secondary fixation carried out in Malta

In terms of classification systems in war trauma we used the Gustilo-Anderson’s classification for open fractures which is widely used mainly due to its simplicity and reproducibility (Table 3) [7], but is perhaps insufficient as sometimes the severity of closed cutaneofascial injury is belied by the extent of the open wound. Alternatives in injury classification include the AO foundation classification of soft tissue injury in open fractures [8, 9] and Red Cross Classification of War Wounds, the latter of which classifies the wound itself [4, 10].

## Case reports

### Case 1

**Fig. 1:**
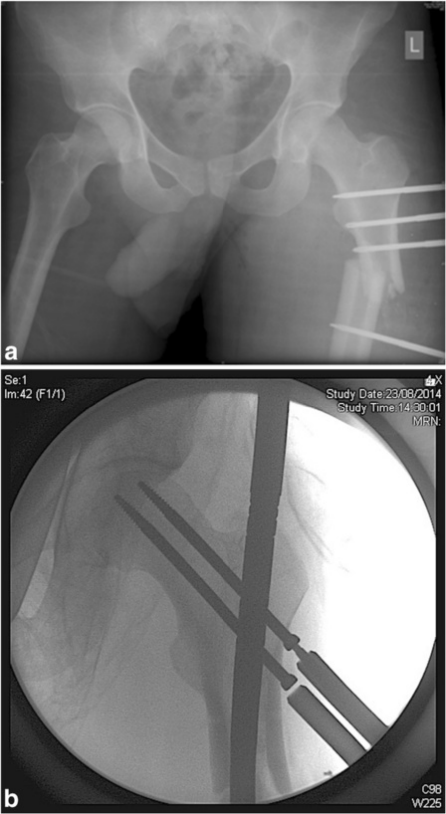
**a** Original DCO external fixator to left proximal femur. **b** Definitive treatment of his left femoral fracture by conversion to a proximal femoral intramedullary nail

Fourty year old gentleman involved in severe IED blast injury a few days prior to arriving in Malta affecting his head, chest and lower limbs. He was haemodynamically unstable, with a IGS2 score of 69. He presented in a state of sepsis and was directly transferred to the intensive care unit. His orthopaedic injuries included an unstable closed left subtrochanteric femoral fracture and an open comminuted fracture of the right tibia and foot with extensive soft tissue disruption graded as a Gustilo-Anderson type IIIC. His other injuries including posterior parietal soft tissue contusion, and right lower limb traumatic vascular injuries to the posterior tibial artery. DCO was performed in Libya where an external fixator was applied to his left femur (Fig. 1a). He then underwent conversion to a left intramedullary femoral nail (Fig. 1b) once stable a few days later. Marginal wound debridement and adjustments to the spanning external fixator was performed to the right leg concurrently. After a trial recovery period postoperatively along with intravenous antibiotics, his right lower leg with the partially missing calcaneum was unfortunately unsalvageable. He subsequently underwent a delayed right below knee amputation two weeks later. After being fitted with a prosthetic leg, his postoperative recovery was stable and he was transferred to a rehabilitation hospital for further care three weeks later.

### Case 2

**Fig. 2:**
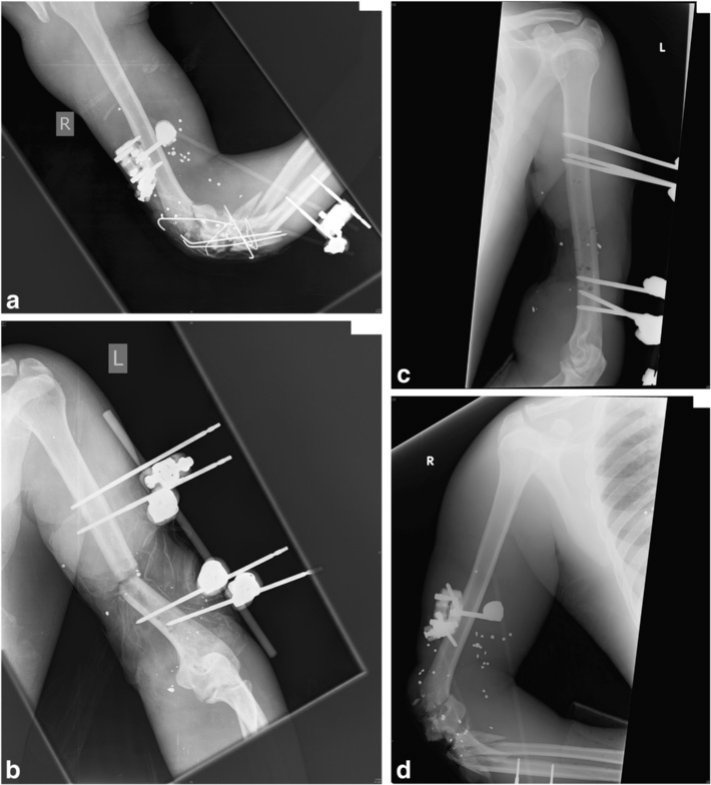
**a** Original DCO external fixator and K wires to the Gustilo-Anderson type IIIB open fracture of right elbow. **b** Original DCO external fixator with complete loss of skin coverage over midshaft left humerus (Gustilo-Anderson type IIIC). **c** Fracture position on revising the alignment of the external fixator. **d** Secondary procedure in Malta. Complete debridement and removal of all metalwork of right elbow except the spanning fixator to maintain stability. Note multiple soft tissue foreign bodies from shrapnel injuries

Fifty year old gentleman involved in an IED blast and GSW. He underwent DCO in Libya and had a spanning external fixator with multiple K wires to stabilise a comminuted right elbow articular fracture (Gustilo-Anderson type IIIB, Fig. 2a). His presented haemodynamically stable with a IGS2 of 46. He also had a non-spanning external fixator applied to his left humerus for an open mid-shaft fracture (Gustilo-Anderson type IIIC, Fig. 2c). Other associated injuries were a complete loss of tissue coverage on the left mid humerus leaving bone exposed. The plastic surgical and vascular surgical teams were also involved in his initial assessment. He was taken to theatres within a few hours of arrival whereby his external fixation was revised, precise wound debridement performed (Fig. 2c), along with concurrent brachial artery bypass using his native greater saphenous vein graft for an brachial artery rupture due to septic erosion. His right elbow required serial debridements every few days and removal of metalwork due to on-going infection (Fig. 2d). After seven debridements and necrectomies over a period of one month along with prolonged intravenous antibiotics, he succumbed to an overwhelming systemic bacteraemia due to Klebsiella pneumonia Carpapenamase (KPC), a highly drug resistance Gram negative bacilli. Prior to the arrival of Libyan war trauma victims, our hospital and country had never had any documented cases of KPC. He died as an inpatient following multi-organ dysfunction and disseminated intravascular coagulopathy.

### Case 3

**Fig. 3:**
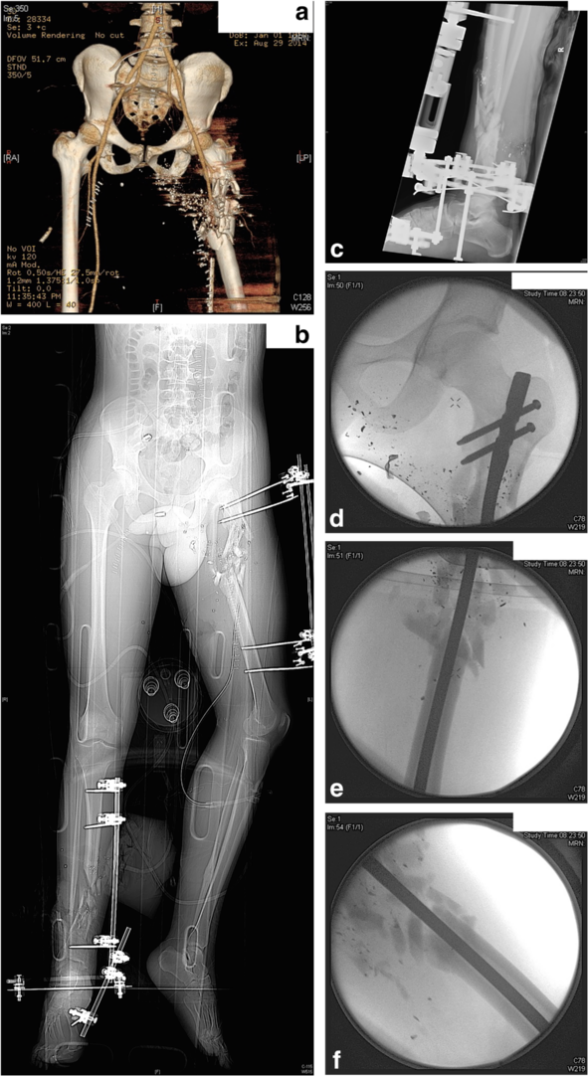
**a** 3D reconstruction showing extent of left comminuted open proximal femur injury. **b** Scout radiograph showing the original DCO procedures to the right tibia and left femur as described in the text. **c** Post-operative view of the right tibia after conversion to a hybrid ring fixator. **d**-**f** Intra-operative radiographs showing the comminuted subtrochanteric femoral fracture initially treated with external fixation but then converted definitively to an intramedullary nail

Thirty two year old man suffered a GSW to the left proximal femur and blast injury to the right leg secondary to an RPG. He underwent DCO initially with application of an external fixator for his left comminuted proximal femoral shaft (Gustilo-Anderson type IIIA). The extent of the comminution is well represented in the CT reconstructions seen in Fig. 3a. He also sustained a right distal tibial diaphyseal shaft fracture (Gustilo-Anderson type IIIB, Fig. 3b). He was haemodynamically stable on arrival, with an IGS2 score of 43. Other injuries included neurological compromise to the left leg with sciatic nerve palsy, bilateral lung contusion, stable superior and inferior pelvic fractures and a stable T12 fracture. Once stable from his lung injuries, the right tibial spanning external fixator was converted to a hybrid ring fixator as seen in Fig. 3c, and the proximal femoral fixator was converted to an interlocking intramedullary nail with bridging of the fracture site (Fig. 3d-f). He made a steady post-operative recovery and was able to mobilise initially non weight bearing then progressed to partial then weight bearing as tolerated with crutches. After eight weeks as an inpatient, he had good bone callous as evidenced in radiographs, along with full functional range of motions of the hip and knee joints. He was deemed fit to be transferred to a local rehabilitation hospital.

### Case 4

**Fig. 4:**
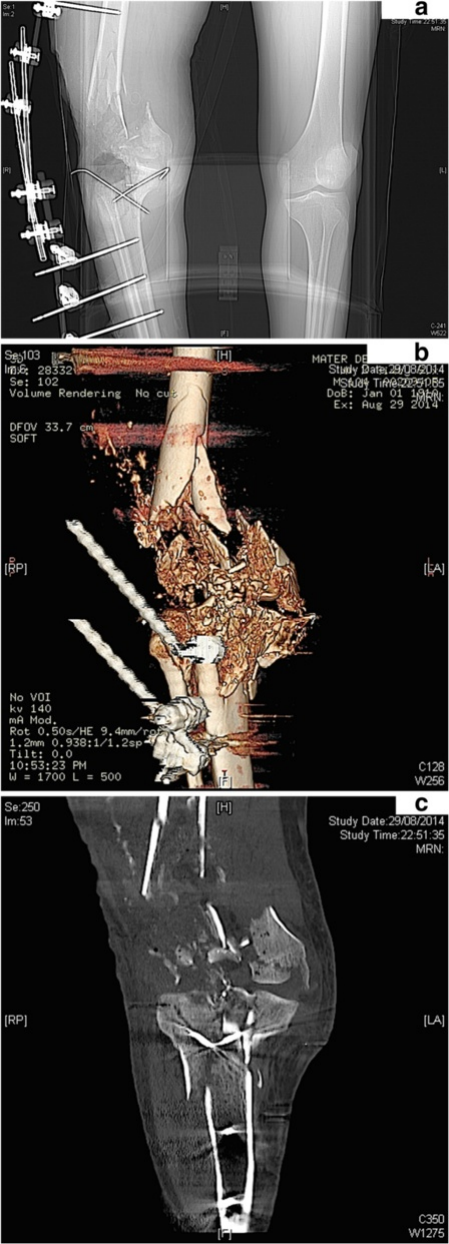
**a** DCO with a spanning external fixator holding what is left of the knee out to length. **b**-**c** CT 3D reconstruction (*above*) and coronal view (*below*) showing extent of knee trauma

Twenty two year old who suffered a GSW to his right knee. He had a comminuted open fracture of his knee, with a shattered the distal femur, patella and tibial plateau as depicted in the reconstructed CT images and extensive soft tissue disruption (Gustilo-Anderson type IIIB, Fig. 4a–c). He had altered neurology in keeping with tibial nerve injury but intact vascular system distal to the knee. His parameters on admission were stable with a IGS2 score of 33. The patient was transferred with a spanning external fixator and multiple K-wires. These were removed in view of skin and joint infection, the wounds debrided thoroughly and the fracture treated definitively with another external fixator. He needed a total of five further debridements and necrectomies in theatres over a span of three weeks. However the patient discharged himself from hospital against medical advice before definitive soft tissue cover could be planned.

### Case 5

**Fig. 5:**
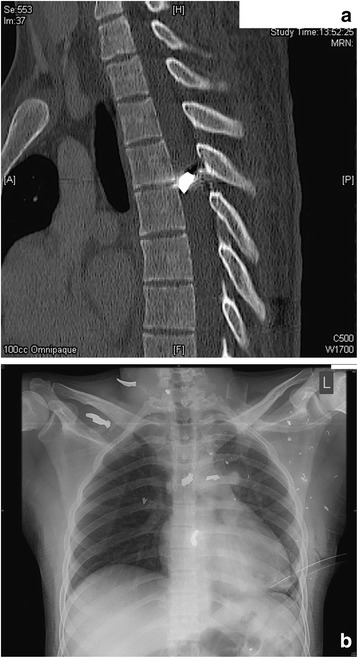
**a** Sagittal CT images showing the metallic foreign body within the spinal canal at the level of T5. **b** Plain chest radiograph showing multiple foreign bodies as typically seen in IED blast injuries

Twenty-six year old man who involved in a blast injury when an bomb exploded near him whilst he was driving his vehicle. He suffered from extensive shrapnel injuries. He was transferred to Malta within 4 days of the incident. He presented with a dense lower limb hemiplegia. A CT scan showed a 1.5 cm sized metallic foreign body at the level of T5 within the spinal canal (Fig. 5a). He also suffered from a left sided pneumothorax that required a chest drain, a fractured right scapula and fractured ribs along with multiple metallic foreign bodies in the chest wall (Fig. 5b). His parameters on admission were stable, with an IGS2 score of 33. Due to the dense paralysis distal to T5 that he presented with, it was deemed that there would be no benefit from removing the foreign body and thus no surgical interventions were performed on the spinal cord. He was also diagnosed with a left popliteal deep venous thrombosis one month into his inpatient hospital stay and was treated medically for this. Physiotherapy together with nursing care were the mainstays of his treatment. He was eventually transferred to a rehabilitation hospital.

### Case 6

**Fig. 6:**
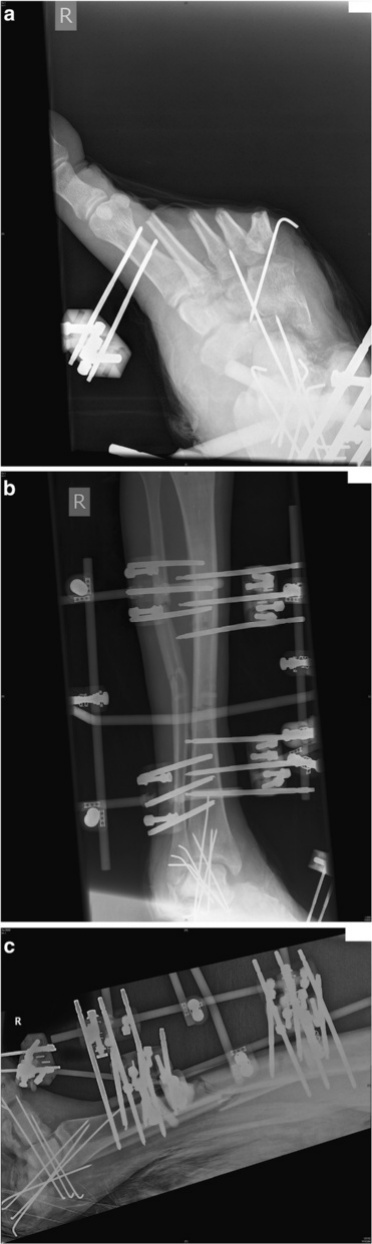
**a** Initial DCO tibio-metatarsal external fixator with amputation of lateral 4 toes through metatarsal bones. **b**-**c** Open tibial and fibular shaft fractures with extensive soft tissue loss

Twenty seven year old man involved in an above ground explosive blast injury that severed the lateral half of his right foot (Fig. 6a). He also had a open tibial and fibular shaft fractures with extensive soft tissue loss (Fig. 6b–c). His parameters at admission were stable, with an IGS2 score of 43. In Libya he underwent DCO with an external fixator applied to the right foot and a metatarsal ray amputation to the lateral four toes. The extent of the soft tissue damage to the leg was significant (Gustilo-Anderson type IIIC) with obvious neurovascular disruption. The plastic surgeons were involved after the initial debridement on admission in an attempt to reconstruct and cover the foot using skin flaps and skin skin grafting, but it was deemed un-salvageable after two plastic surgical procedures. He required two further soft tissue debridements, however an eventual right below knee amputation was performed after four weeks. He made a good recovery and was then transferred to a rehabilitation hospital.

## Discussion

Our hospital received in excess of one hundred and fifty Libyan civil war casualties between June 2014 and January 2015, most of whom were treated by our orthopaedic department at Mater Dei Hospital, Malta. Our hospital has a bed capacity for 925 patients. We perform an average of 1800 civilian orthopaedic trauma operations per annum.

We received patients from a wide range of Libyan cities and outskirt towns from both public and private hospitals. They presented with extensive bony and soft tissue injuries, soft tissue infection and necrosis, as well as haemodynamically unstable patients largely due to combination of the severity of their injuries and prolonged evacuation and transit. Our case series echoes current anatomical trauma patterns seen with injuries caused by war, especially those caused by IEDs which are designed to destroy and incapacitate personnel and vehicles [3].

Our role as a civilian tertiary hospital turned to that of a Level 1 Trauma hospital was our first experience as a hospital and unit in dealing with an influx of war trauma casualties on a daily basis. It not only put a strain on the National Health Service, but also on individual departments including intensive care, operating theatres, surgery and orthopaedics/trauma. As an orthopaedic department we aimed to treat the injuries definitively, converting to internal fixation when permissible in line with DCO, restoring functional mobility and curtailing soft tissue and joint infections.

The usage of the term ‘damage control surgery’ has gained popularity since the mid 1990’s [11], however its principles have been alluded to in various literature from the Napoleonic campaigns in 18th century, through to major world wars in the 19th and 20th century.

The phrase “damage control” is traditionally a navy term. It refers to keeping a badly damaged ship afloat after major penetrating injury to the hull. Procedures for temporary righting and stabilising the ship, which keep the ship afloat, permit assessment of other damage and time to establish a sensible plan for definitive repair. The analogy to care of the seriously injured trauma patient is likened to this concept [5, 12].

Damage control surgery was initially practised by general surgeons by packing the abdominal cavity to control diffuse bleeding from solid organs and other structures [12], thus preventing the lethal triad of coagulopathy, acidosis and hypothermia [13]. Damage control surgery consists of three phases: first, the control of haemorrhage and contamination; secondly, rewarming and correction of coagulopathy; and thirdly, surgical re-exploration and definitive repair [13]. DCO is an extension of damage control surgery [2]. It comprises early marginal and meticulous wound debridement, temporary fracture stabilisation typically through the use of an external fixator, minimal blood and heat loss, physiological stabilisation, and then secondary definitive orthopaedic management after medical evacuation [2, 14–16].

If we consider, from a physiological point of view, the aetiology of the war injuries as the patient's “first hit”, the purpose of DCO is to avoid worsening the patient’s condition by the “second hit” of a major orthopaedic procedure and to delay definitive fracture repair until the patient's general physiological condition is optimised. The second hit phenomenon has added systemic physiological effects affecting morbidity and mortality by exhausting a patient's biological reserve [11]. Definitive open reduction and repair is delayed until the inflammatory response and tissue oedema has decreased and the patients are clinically stable [13]. The incidence of multiple organ failure decreased significantly from the times of early trauma care to the DCO period regardless of the type of treatment of the femoral fracture thus proving of effectiveness of the current practise of DCO [14, 16].

In conjunction with DCO comes the current challenge of infection prevention. Injuries from IEDs differ markedly from GSWs. The contamination and soft tissue injury require more aggressive treatment [4, 6]. IEDs come in forms of buried artillery rounds, above ground explosives, and car bombs amongst others [6]. Other forms include mortars, rockets, and RPGs [2]. Wounds should not be closed primarily but rather debrided thoroughly and covered temporarily. Splinting and external fixation are mainstays of bony stabilisation [2].

Our experience as a tertiary centre and Level 1 care hospital was an extension of the DCO strategy. Our efforts to convert external fixators to definitive internal fixation within a timeframe of two weeks were greatly hampered and at times deemed impossible by high levels of systemic sepsis, local soft tissue infection and osteomyelitis. The majority of cases had open fractures which are known risk factors for bony non-union and prosthesis failure [10, 11].

Scoring systems such as the Gustilo-Anderson classification (Table 3) correlates the severity of the fracture and soft tissue injury to the rate of infection and thus has prognostic value [7]. Gustilo et al. [17] presented their own experience with Type III injuries showing wound sepsis in the three subtypes were: Type IIIA, 4 %, IIIB, 52 %; and IIIC, 42 %; while amputation rates were, respectively, 0 %, 16 %, and 42 %. Our experience mirrored their results with the majority of fractures in this case series being most severe in the Gustilo-Anderson classification, scoring Type IIIB and Type IIIC.

**Table 3:** Gustilo and Anderson classification of open fractures [7]

Type I	Open fracture with laceration <1 cm and clean
Type II	Open fracture with laceration >1 cm without extensive soft tissue damage, flaps of avulsions
Type III	Open segmental fracture with >10 cm laceration with extensive soft tissue injury or traumatic amputation. Any gunshot injury or farm machinery injury falls into this category. Type III are further subdivided into three categories (A, B and C).
IIIA	Adequate soft tissue overage
IIIB	Significant soft tissue loss with exposed bone that requires tissue transfer to achieve bony coverage
IIIC	Associated vascular injury that requires repair for limb preservation

We underline the difficulties of repeated planned limb reconstruction procedures required in order to attain satisfactory functional results. There was difficulty persuading victims of war zones for followup procedures as shown in our case 4 in this series.

The types of original fixation we encountered by and large stayed true to the principles of DCO by being monoplane and evolutive, with small number of pins placed distant to the fracture site with the aim of reducing the incidence of fracture site infection that could compromise later definitive treatment [5].

Secondary internal fixation remains a controversial issue in management of battlefield injuries. Both Murray et al. in 2008 [3] and Mody et al. in 2011 [18] reported a 40 % infection rate, with up to 17 % osteomyelitis. Infections occurred secondary to blast injuries in 91 % of cases. Furthermore, Murray [3] reported that intramedullary nailing is indicated in initially closed fractures as well as open femoral fractures when soft tissue management has allowed proper bone coverage without early infection, and interestingly Mody [18] reported good long term functional results from secondary femoral and tibial nailing despite high rate of infectious complications.

Late conversion from an external fixator to internal fixation is associated with a high risk of infection, with a timeframe of two weeks being the benchmark [19]. This is supported by Mathieu [5] who showed that early conversion to internal fixation for closed diaphyseal fractures yield better results.

The mainstay of secondary definitive treatment carried out by our centre was based on the principles of adequate soft tissue debridement, definitive fracture stabilisation often employing the principle of relative stability by bridging the often comminuted fractures, and then soft tissue cover.

## Conclusion

Our department applied current trends in war trauma orthopaedic treatment and a continuum of the damage control orthopaedic strategy by converting to definitive treatment where permissible. Most were classified as having Type IIIB/C) injuries according to the Gustilo-Anderson classification. This is linked to wound sepsis and to a poor prognosis in terms of limb loss. We treated these infections with repeated debridements and necrectomies and antibiotics according to bacterial sensitivities. The wounds were debrided until it was deemed safe to convert to definitive internal fixation. The injuries treated reflected the type of weaponry available in modern warfare affecting both militants and civilians alike, being of increased severity and with increased body regions involved. The vast majority of cases that were managed at our centre recovered well once definitely operated upon and went on to be transferred to rehabilitation hospitals to continue their rehabilitation.

## Consent

Verbal consent was gained from all patients for publication of this case series and any accompanying images. Written consent was deemed invalid since all patients were anonymized upon arrival to our institution to protect their identities.

## Abbreviations

AO: Arbeitsgemeinschaft fur Osteosynthesefragen
CT: computer tomography
DCO: damage control orthopaedics
GSW: gun shot wound
IED: improvised explosive device
IGS2: indice de gravité simplifié 2
KPC: Klebsiella pneumonia Carbapenamase
RPG: rocket propelled grenade
